# A large consanguineous family with a homozygous Metabotropic Glutamate Receptor 7 (mGlu7) variant and developmental epileptic encephalopathy: Effect on protein structure and ligand affinity

**DOI:** 10.1186/s13023-021-01951-w

**Published:** 2021-07-17

**Authors:** Marwa Ben Jdila, Cécile Mignon-Ravix, Sihem Ben Ncir, Fatma Kammoun, Faiza Fakhfakh, Laurent Villard, Chahnez Triki

**Affiliations:** 1 Research Laboratory ‘NeuroPédiatrie’ (LR19ES15), Sfax Medical School, Sfax University, Sfax, Tunisia; 2 Laboratory of Molecular and Functional Genetics, Faculty of Science of Sfax, Sfax University, Sfax, Tunisia; 3 Inserm, MMG, Aix Marseille Univ, Marseille, France; 4 Child Neurology Department, Hedi Chaker Universitary Hospital of Sfax, Sfax, Tunisia; 5 Département de Génétique Médicale, Hôpital d’Enfants de la Timone, Assistance Publique Hôpitaux de Marseille, 13385 Marseille, France

**Keywords:** Developmental epileptic encephalopathy, *GRM7* gene, Next generation sequencing

## Abstract

**Background:**

Developmental and epileptic encephalopathies (DEE) are chronic neurological conditions where epileptic activity contributes to the progressive disruption of brain function, frequently leading to impaired motor, cognitive and sensory development.

**Patients and methods:**

The present study reports a clinical investigation and a molecular analysis by Next Generation Sequencing (NGS) of a large consanguineous family comprising several cases of developmental and epileptic encephalopathy. Bioinformatic prediction and molecular docking analysis were also carried out.

**Results:**

The majority of patients in our studied family had severe developmental impairments, early-onset seizures, brain malformations such as cortical atrophy and microcephaly, developmental delays and intellectual disabilities. The molecular investigations revealed a novel homozygous variant c.1411G>A (p.Gly471Arg) in the *GRM7* gene which was segregating with the disease in the family. Bioinformatic tools predicted its pathogenicity and docking analysis revealed its potential effects on mGlu7 protein binding to its ligand.

**Conclusion:**

Our results contribute to a better understanding of the impact of *GRM7* variants for the newly described associated phenotype.

## Introduction

Epileptic Encephalopathies (EE) are a group of heterogeneous epileptic syndromes associated with severe cognitive stagnation or regression and behavioral disturbances due to frequent epileptiform activity [1]. The cognitive and behavioral impairments are caused by the epileptic activity itself above and beyond what might be expected from the underlying pathological one [1]. Many epileptic encephalopathies are known to have an identifiable molecular genetic basis. The genetic cause often leads to developmental delay on its own, with epilepsy worsening the development. In the latest ILAE classification, the term was changed to developmental encephalopathies and epilepsy (DEE) [2].

Recent advances in high-throughput parallel sequencing technologies allowed the identification of variants in more than 100 genes associated with DEE. Those encoding synaptic proteins include AMPA ionotropic receptor GluA2 subunit [3] and glutamate receptor ionotropic NMDA Type subunit 1(GRIN1) [4], subunit 2A (GRIN2A) [5], or subunit 2B (GRIN2B) [6] and recently the *GRM4* and *GRM7* genes encoding metabotropic glutamate receptor 4 (mGlu4) and 7 (mGlu7), respectively [7, 8]. The mGlu receptors are G-protein coupled receptors that modulate neurotransmission and synaptic plasticity throughout the central nervous system [9]. Particularly, the mGlu7 protein is a GTP-binding protein–coupled receptor (GPCR) with a heterodimeric structure exclusively expressed in the central nervous system (CNS) with a relatively high expression in the cortex, amygdala, hippocampus, and hypothalamus [10, 11]. The mGlu7 dimer contains two large extracellular domains called the Venus flytrap domains (VFD) containing the glutamate-binding site and cysteine-rich domains (CRDs), seven transmembrane-spanning domains called heptahelical domains (HD) and a C-terminal intracellular domain. Conformational changes induced by ligand binding to the mGlu7 allowed the propagation of signals from the VFD via CRDs to the HD domain and the C-terminal tail [9]. Indeed, mGlu7 plays a critical role in synaptic transmission in neurons where it can act as an auto- or hetero-receptor by inhibiting further release of excitatory neurotransmitter glutamate and inhibitory neurotransmitter GABA, respectively [9, 12, 13].

Here, we describe the clinical and molecular findings in a large consanguineous Tunisian family comprising several cases of DEE. We identified a novel homozygous missense variant in the *GRM7* gene segregating with the disease in all tested individuals. Bioinformatic tools and docking analysis were performed to predict the effect of the variant on the protein function.

## Patients and methods

### Patients

**Fig. 1:**
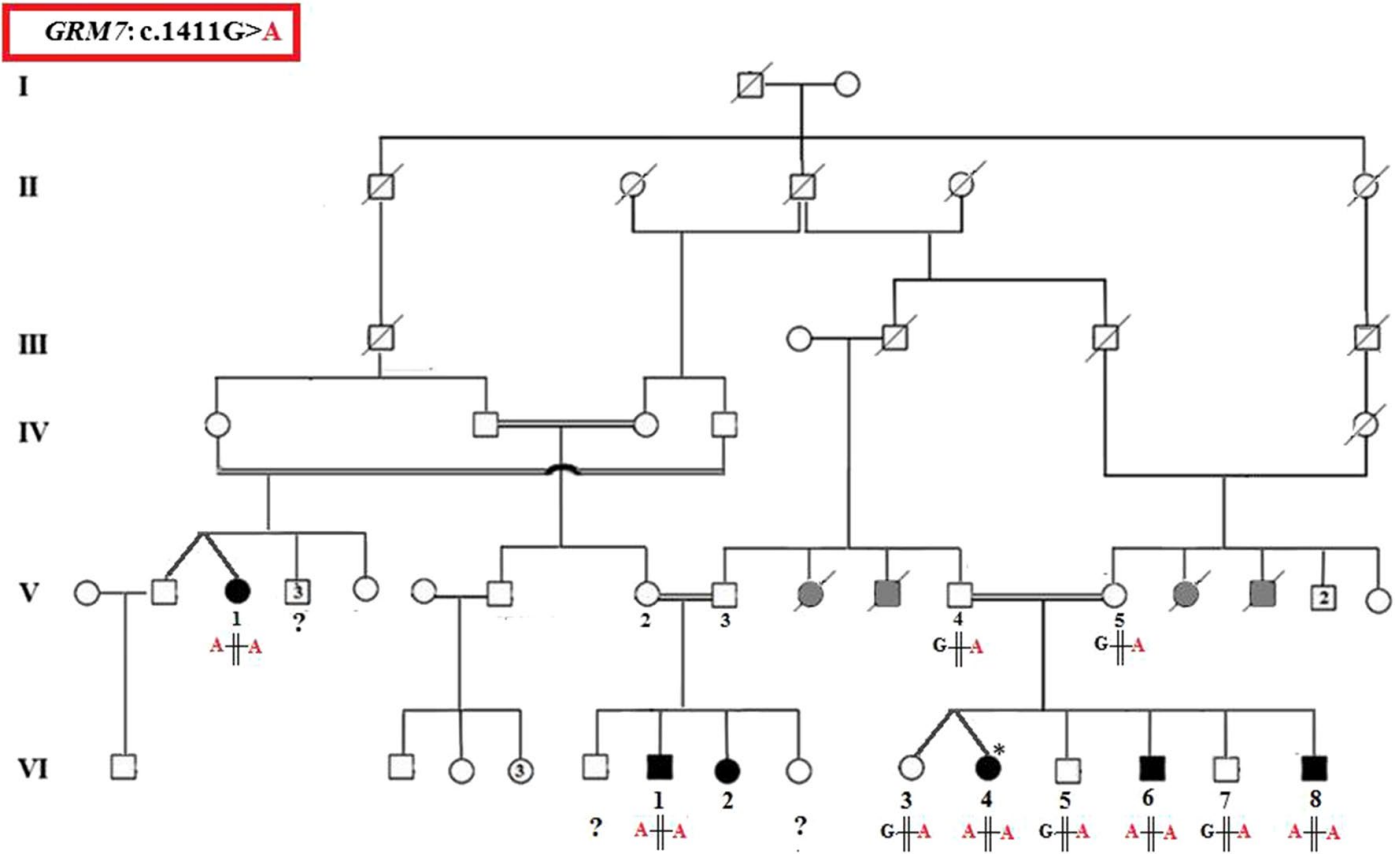
Pedigree of the large studied family with EE presenting the segregation of the *GRM7* variant. Asterisk indicates the patient with West Syndrome whose DNA sample was sequenced using the TruSight One Sequencing Panel. Black shapes mark affected individuals. Gray shapes indicate those have not released medical records but the family informed us that they have a neurological disorder

This study was carried out on a large consanguineous Tunisian family with 6 individuals being affected by severe epilepsy (Fig. 1). Two affected children (VI1 and VI4) were followed in the Child Neurology Department of Hedi Chaker Hospital in Sfax (Tunisia). Magnetic resonance imaging (MRI) was performed only for these two patients. Affected and unaffected individuals were thoroughly examined by child neurologist and genetic consultants during an onsite visit. Family medical history and the consanguineous relationships of the parents were obtained by interviewing family elders and guardians. All information was cross checked by interviewing relatives. Informed consent was obtained from all individuals involved in the study or their legal representatives for genetic study and publication of photos. The study was performed in agreement with the ethical standards of the local ethics committee.

### DNA extraction

Peripheral blood samples were collected from the affected individuals (V1, VI1, VI4, VI6 and VI8), normal parents (V4, V5), and siblings (VI3, VI5 and VI7). Total DNA was extracted from peripheral blood using phenol chloroform standard procedures [14].

### Next generation sequencing (NGS)

A DNA sample from VI1 was sequenced using the TruSightOne Sequencing Panel (Illumina, Inc., San Diego, CA, USA) on a NextSeq 500 sequencing apparatus. Sequencing data were analyzed according to an autosomal recessive transmission mode (i.e. homozygous or compound heterozygous). We selected the variants affecting the protein sequence and removed variants present with a minor allele frequency > 0.001 in the GnomAD v2.1 database (http://gnomad.broadinstitute.org/). We kept the variants predicted to be pathogenic or likely pathogenic by at least three prediction software among the five used routinely in our analyses (UMD predictor http://umd-predictor.eu/, SIFT http://sift.bii.a-star.edu.sg/, Polyphen-2 http://genetics.bwh.harvard.edu/pph2/, LRT and Mutation Taster http://www.mutationtaster.org/). Finally, we removed the variant located in genes not expressed in the brain after query of the Genotype-Tissue Expression (GTex) portal (https://www.gtexportal.org/home/).Validation and segregation analysis of the *GRM7* variant was done using Sanger sequencing with the following primers: GRM7-F**:**TAAGTCTCTAGCCTGTCACC and GRM7-R: GATATCAGTTCCTGCTGATG.

### Prediction of protein stability

I-Mutant 2.0 is a SVM-based method for the automatic prediction of protein stability changes upon single-site mutations. The output file shows the predicted free energy change (DDG). This value is calculated from the unfolding Gibbs free energy change of the mutated protein minus the unfolding free energy value of the native protein (Kcal/mol) [15].

### Sequence alignment and prediction of 3D protein structure

The evolutionary conservation of the altered amino acid was investigated using the Clustal W algorithm (www.Ebi.ac.uk/tools/clustalw2/). The structural effects were predicted using the SNP effect 4.0 database. Predictions included four properties of the protein to note aggregation tendency (TANGO), amyloid propensity (WALTZ), chaperone binding (LIMBO) and protein stability (FoldX). For each property, the difference of score between normal and mutated structure was calculated to evaluate a possible alteration [16]. To understand the effect of the non-synonymous variant changing a Glycine to an Arginine at position 471 (p.Gly471Arg) in the extracellular ligand binding domain of the mGlu7 protein structure, we modeled and compared the two variants, 471G and 471R. We used PSI-BLAST to select the best template ‘‘5c5c.1” PDB structure, with a homology of 50% of the mGlu7 protein sequence. “5c5c.1” is a structure of the human metabotropic glutamate receptor 7 extracellular ligand binding domain. The generation of the two theoretical 3D models was achieved by the MODELLER9 v8 software [17]. The SWISS PDB VIEWER software (V4.1) was used to display and compare models. The quality of the models was evaluated using ProSA-web [18].

### Molecular docking of the Glutamate ligand at the mGlu7 protein binding site

Before the docking, the Glutamate molecule was generated using the ChemBio3D Ultra 12.0 software (CambridgeSoft Co., USA) and its energy was minimized with the MM2 tools implemented to the software. The molecular docking of the chemical compounds at the mGlu7-binding site was performed using the AutoDock Vina software [19]. The docking runs were carried out with a radius of 40A˚ with coordinates x: 15.167, y: 4.556 and z: 15.611. The best-ranked docking pose of each chemical compound in the active site of mGlu7 was obtained according to the scores and binding-energy value. Ligand-enzyme interactions were analyzed and drawn by using the Biovia Discovery Studio Visualizer developed by Accelrys (BIOvIA, D. S. (2016). Discovery studio modeling environment, San Diego, Dassault Systemes, Release, 4.). After the best docking pose was chosen, the major effect of the mutated mGlu7 protein in ligand binding was visualized using Pymol.

## Results

### Clinical findings

**Fig. 2:**
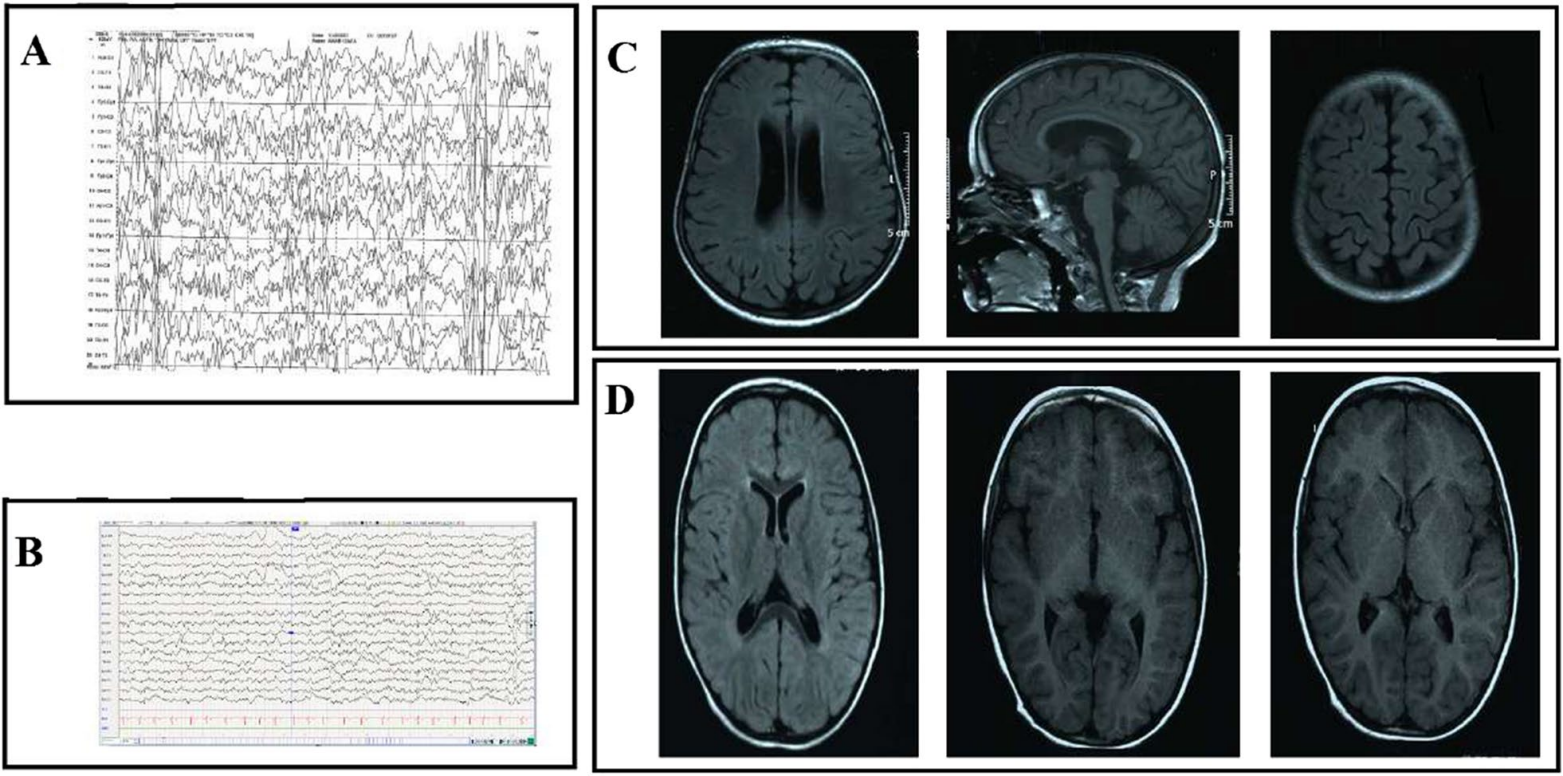
**a** Sleep (EEG) showing an aspect of hypsarrhythmia: high amplitude and irregular waves and spikes in a background of chaotic and disorganized activity. **b** Awake EEG showing multifocal spikes discharges. **c**, **d** Coronal section of brain MRI from patients carrying the p.Gly471Arg mGlu7 variant. **c** Coronal section of brain MRI of VI4 patient at the age of 10 years showed a cortical and sub-cortical atrophy. **d** Coronal section of brain MRI of VI1 patient at the age of 2 years showed a discrete hyper T2 of posterior SB

**Fig. 3:**
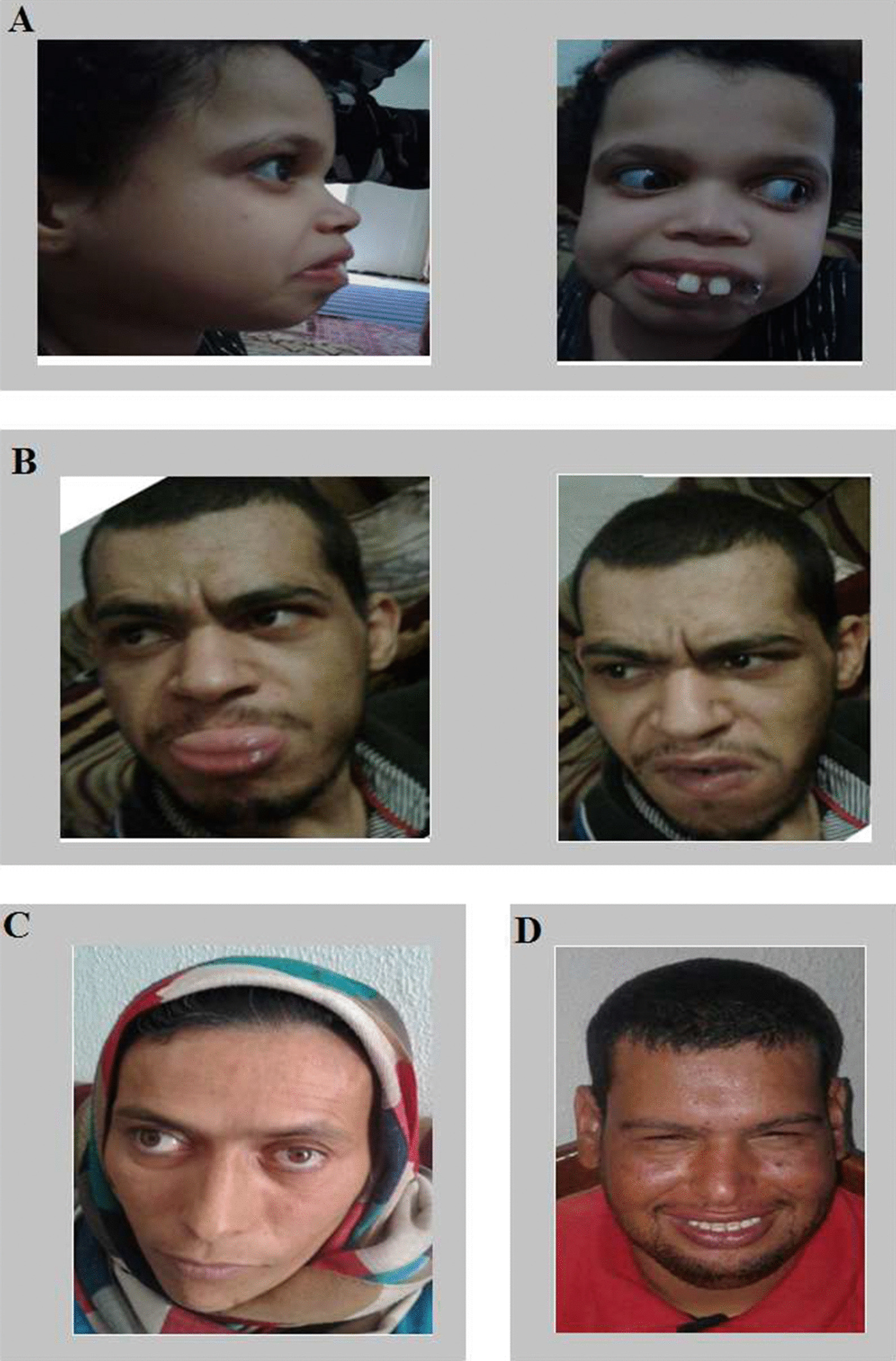
Photographs showing dysmorphic features of affected family members: VI4 (**a**), VI8 (**b**), V1 (**c**) and VI6 (**d**). (Photographs reproduced with patients’ permission)

The index case (VI4) presented a developmental and epileptic encephalopathy. She was born at term after normal pregnancy and delivery. Birth weight and head circumference were normal. She had a motor and cognitive delay. At the age of 3 months, she developed focal to bilateral clonic seizures. She was then diagnosed at 6 months with West syndrome (Fig. 2a) which evolved to Lennox Gastaut syndrome (Fig. 2b) at the age of 3 years. She was treated by several anti-seizure drugs (vigabatrin, valproic acid, Benzilate, lamotrigine). She had no seizures since the age of 11 years. At the age of 10 years, her EEG showed an abnormal background with multifocal epileptiform discharges. Currently 13 years old, she is bedridden with profound global developmental delays and intellectual disability, no language, spastic tetraplegia and hand stereotypies. She has a marked dysmorphic face with a broad forehead, globular eyes and convergent strabismus, a wide mouth with prominent and spaced teeth and an everted lower lip (Fig. 3a). She acquired microcephaly and scoliosis. An on-site investigation revealed several relatives, including 2 brothers (VI6 and VI8), cousins (VI1, VI2) and a distant aunt (V1). For all these persons, pregnancy and delivery were normal. They all had seizures since the infancy (between 3 months and 1 year). It was difficult to determine the type of seizures that these people experienced during infancy, as their parents did not remember the semiology of the seizures; but they all described seizures as generalized tonic-clonic or focal motor during childhood and adolescence (Table 1). All these persons had a severe delay of motor and cognitive development with intellectual deficiency and stereotypies. Facial dysmorphy was more marked in the index case, her brothers (VI6, VI8) had coarse faces with wide mouths (Fig. 3b, d). Metabolic screening for the index case was normal. MRI was performed for her (VI4) and her cousin (VI1) and revealed non-specific abnormalities with cortico-subcortical atrophy (Fig. 2c, d). Available clinical information is summarized in Table 1.

**Table 1:** Clinical features and comparative data of affected individuals of the studied family

	VI4	VI6	VI8	VI1	VI2	V1
Age (years)	13	32	30	9	7	41
Gender	F	M	M	M	F	F
Epilepsy						
Age at onset of seizure	3 months	1 years	1 years	16 months	1 years	ND
Seizure type	Epileptic spasm	Generalized tonic-clonic seizures	Generalized tonic-clonic seizures	Focal seizures Tonic seizures	Generalized tonic-clonic seizures	Generalized tonic-clonic seizures
Persistent epilepsy	No seizure since the age of 11 years	Yes	Yes	No	Yes	Yes
Intellectual disability	Profound	Profound	Profound	Profound	Profound	Profound
Microcephaly	Yes (-2DS)	ND	ND	ND	ND	ND
Langage	Absence	Absence	Absence	Absence	Absence	Language disorder
Psychomotor development	Delay	Delay	Delay	Delay	Delay	Delay
Stereotypies	Yes	Yes	Yes	Yes	Yes	Yes
Behavioral disorders	Bruxism Gestural stereotypy: (stereotypy of the head, hands in the mouth, washing)	Hetero-aggressive reaction Onychophagy	Verbal stereotypy agitation Self-aggression Hetero-aggressivity Gestural stereotypy (tapping, sway of the anterior-posterior trunk, washing)	Agitation Self-aggression Hetero-aggressivity Gestural stereotypy (tapping, sway of the anteroposterior trunk, washing)	Verbal stereotypy Agitation Self-aggression Hetero-aggressivity Gestural stereotypy (tapping, sway of the anterior-posterior trunk)	Hetero-aggressive
Facial dysmorphism (Fig. 3)	Frontal hump, convergent strabismus of the eye, globular eyes, incisors large and prominent, prominent upper dental arch	Wide mouth, small eyes	Wide mouth, macroglossia	ND	ND	Large globular eyes, convergent strabismus
MRI (Fig. 2c, d)	Cortical and subcortical atrophy and thin corpus callosum (at the age of 3 years)	ND	ND	Discrete hyper T2 of posterior SB (at the age of 2 years)	ND	ND

ND, not determined; F, female; M, male

### Genetic analysis, structural modeling and molecular docking

The genomic DNA of patient VI4 was sequenced using a clinical exome sequencing kit. The results revealed the presence of a novel homozygous variant in the *GRM7* gene (c.1411G>A, NM_000844, p.Gly471Arg). The presence of this variant was confirmed by Sanger sequencing and it was shown to be homozygous in affected individuals V1, VI1, VI4, VI6 and VI8, and heterozygous in the tested unaffected family members V4, V5, VI3, VI5 and VI7 (Fig. 1).

**Fig. 4:**
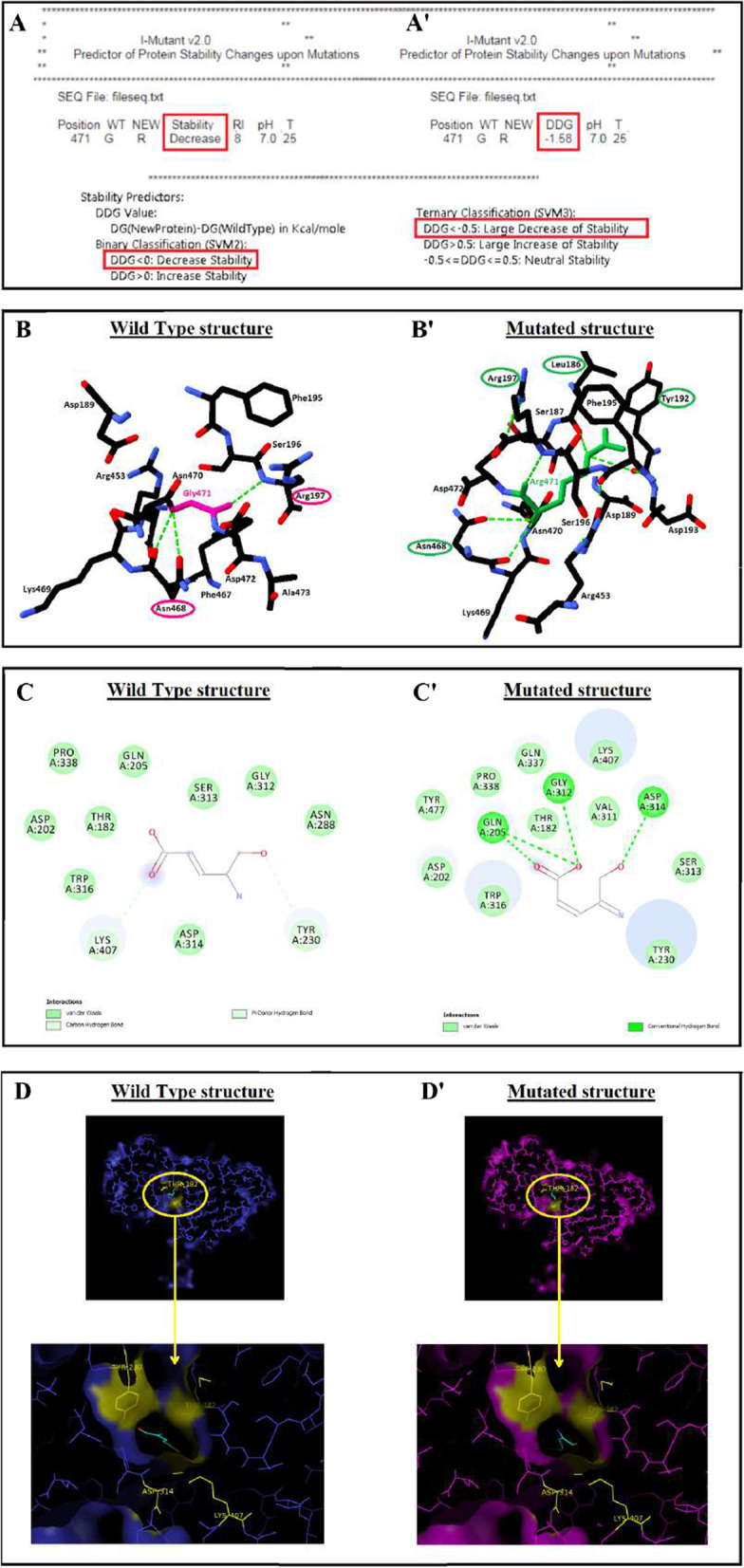
**a–a**′ Prediction of the functional effect of p.Gly471Arg variant by Mutation Taster. **b–b**′ Differences in hydrogen bond connections between wild-type and mutated models: Gly471 establishes three hydrogen bonds with Arg197 and Asn468 (**b**), the Arg471 variant leads to an addition of two new hydrogen bonds with Leu186 and Tyr192 (**b**′). The wild type amino acids are colored in pink, the variant amino acid is colored in green. **c–c**′ Glutamate (Glu) recognition by mGlu7 protein. Hydrogen atoms attached at the C_α_ atom of the ligand (Glu) are modeled with the corresponding ideal geometries. Dark green and light grey lines indicate hydrogen-bonding while light green lines indicate VDW contact. **d–d**′ The ligand-binding pocket. The orientation of Glutamate docked into the putative active site of mGlu7. **d** The structure of wild-type mGlu7. (**d**′) The structure of the Gly471Arg mutant

The c.1411G>A transition substitutes the highly conserved Glycine 471by an Arginine residue in the VFTD (Venus Flytrap sub-domain) of the N-terminal domain of mGlu7. The I-Mutant program predicted that this variation would largely decrease the stability of mGlu7 protein (Fig. 4a–a′). To verify these results and to look for a potential change in 3D protein structure, we compared the normal and mutated 3D models of mGlu7 protein (Fig. 4b–b′). The 3D Model revealed that Gly471 established three hydrogen bonds with Arg197 and Asn468. The Arg471 variation led to the addition of two new hydrogen bonds with the residues Leu186 and Tyr192 that may affect the spatial conformation of the protein (Fig. 4b–b′).

This conformational change in the mGlu7 protein could affect the general organization of the dimer forming the ligand-binding region. To evaluate the effect of structural changes of VFTD domain on the ligand binding, we performed the mutated mGlu7 protein docking with its ligand (Fig. 4c–d′). A structural comparison of wild-type and mutant protein suggests that they recognize the glutamate ligand differently. Figure 4c–c′ illustrated the ligand-binding pocket with the conserved residues of wild-type and mutated mGlu7 proteins. Under normal conditions, Hydrogen atoms attached at the Carbon atom of the ligand are connected by hydrogen-bonding and Van DerWaals (VDW) contact with residues belonging not only to the LB1 domain (T182 and k407) but also to the LB2 domain (Y230 and D314) of the mGlu7 receptor. Compared to the wild type, the variant can cause larger conformational fluctuations of ligand and the instability to the key active-site residues. The hydroxyl group of ligand could lose the hydrogen bonds with Y230 and K407 and make new contacts with surrounding residues (Fig. 4c–c′). Besides, the structural analysis of the large cavity forming the ligand binding site observed through PyMOL (Fig. 4d–d′) showed that due to G471R mutation, the ligand was completely buried inside the binding pocket of the mutated mGlu7 protein.

## Discussion

We describe the identification of a novel homozygous c.1411G>A (p.Gly471Arg) variant in the *GRM7* gene in a large Tunisian consanguineous family diagnosed with developmental and epileptic encephalopathies (DEE).

The *GRM7* gene encodes the mGlu7 receptor belonging to mGlu's type III receptors, which are G-protein coupled receptors that modulate neurotransmission and synaptic plasticity throughout the central nervous system. Indeed, recent studies showed that mGlu's type III including mGlu4 and mGlu7 receptors are associated with neurodevelopmental disorders. De novo duplication in *GRM4* gene was found in a patient with a severe psychomotor retardation, epilepsy, mild dysmorphic features and behavioral disturbances [20]. The *GRM4* gene variants were also described as associated with juvenile myoclonic epilepsy, characterized by myoclonic jerks, absence and generalized seizures [21, 22]. Nevertheless, pathogenic *GRM7*mutations were reported in patients diagnosed with neurodevelopmental disorders [23–25]. Recently, Marafi et al. described biallelic variants of *GRM7* in 11 patients belonging to six families with developmental and epileptic encephalopathy (DEE). Most patients in their cohort had a severe developmental impairment, early-onset seizures and frequent abnormal epileptiform activity revealed by EEG and severe neurological phenotype. Based on the clinical presentation of our patients and those described by Marafi et al. (Table 2), we noticed similar features including an early onset of seizure, microcephaly and cortical atrophy, developmental delay and intellectual disability [7]. Epilepsy was polymorphic and could be generalized or focal with age-dependent electro-clinical syndromes (West syndrome then evolves into a Lennox Gastaut syndrome for the index case). For all the family members and 6/9 patients from the cohort studied by Marafi et al., epilepsy is drug resistant. Two of our patients (index case and case VII) had no seizure after the first decade but with a worsened cognitive deficit and behavioral disorders. All these elements are consistent with the diagnosis of developmental encephalopathy and epilepsy. Serial EEG showed a slowing background with focal or multifocal epileptiform activity and a less frequently generalized slow spike and slow wave pattern ([7]; Tables 1, 2). Stereotypes of the hands are neither constant nor specific. Facial dysmorphia was present in only one patient and was similar to that in our index patient. Brain imaging was also nonspecific in the patients described by Marafi et al. where most frequent signs were cortical atrophy, hypomyelination and hypoplasia of the corpus callosum, whereas MRI results showed cortical and sub-cortical atrophy, a thin corpus callosum without white matter abnormality in the index case of the studied family but hypomyelinisation was noticed for her cousin.

**Table 2:** Clinical features and comparative data of our index case with the patients described by Marafi et al. [7]

Reference	Marafi et al. 2020 [7]											Our study
Mutation	p.I154T		p.R658W	p.T675K	p.W586*		p.R658Q			p.E891K	p.R659*	p.G471R
Domain	Ligand-binding domain		Transmembrane domain		Transmembrane domain		Transmembrane domain			Intracellular domain	Trans-membrane domain	Ligand-binding domain
Sex	M	M	F	M	F	M	M	M	F	M	M	F(VI4)
Current age	13 yrs	10 yrs	15 yrs	10 yrs	Died at 13 yrs	N/A	Died at 13 mo (respiratory failure)	Died at 45 days	Died at 4 yrs	2 yrs 2 mo	Died at 5 yrs	13yrs
Age at last Exam	13 yrs.	10 yrs.	15 yrs.	10 yrs.	N/A	5 yrs. 6 mo.	13 mo.	1 mo.	3 yrs. 3 mo.	7 mo.	20 mo.	13 yrs.
Occipital frontal circumference-last exam cm (z score)	50 cm (−2.9sd)	N/A	50.5 cm (−3.31sd)	48.6 cm (−3.29sd)	N/A (<−2 sd)	N/A (<−2 sd)	N/A	N/A	42.5 (−3.8 sd)	40 cm (−3.2 sd	44.5 cm (−2.7 sd)	49 .5cm (−2sd)
Microcephaly	+	N/A	+	+	+	+	N/A	N/A	+	+	+	+
Axial Hypotonia	+	+	+	+	N/A	N/A	+	N/A	+	+	+	+
Peripheral hypertonia	+	+	+	+	N/A	+	N/A	N/A	−	+	+	+
Hyper-reflexia	+	+	−	N/A	N/A	N/A	N/A	N/A	−	+	+	+
DD/ID	+	+	+	+	+	+	+	+	+	+	+	+
Seizures (onset)	+ (4 mo.)	+ (8 mo.)	+ (1 mo.)	+ (1date of life)	+	+ (3 w)	+ (3 mo.)	+ (1 w)	+ (5 mo.)	+ (2 mo.)	+ (2d)	+ (3 mo.)
Drug resistant epilepsy (current anti -epileptic drugs	−	−	+	+	N/A	N/A	+	−	+	+	+	+
Seizure types	Myoclonic	GTCS	Myoclonic & GTCS	Myoclonic & GTCS	N/A	N/A	N/A	N/A	GTCS & focal	Myoclonic & GTCS	Multifocal	Focal tonic, epileptic spasm
Status epilepticus	−	−	+	−	N/A	N/A	+	N/A	−	+	+	−
Electro-encephalogram (EEG) findings Epileptiform activity	**+**	N/A	−	+	N/A	N/A	+	−	+	+	+	+
Type of neuroimaging (age)	Brain MRI (3 yrs)	Brain MRI (2 yrs)	Serial brain MRIs (2 mo, 18 mo, 3 yrs, 7 yrs & 10 yrs)	Serial brain MRIs (2 yrs & 5 yrs)	N/A	Brain MRI (N/A)	CT head (2 mo)	Brain MRI (2 w)	Brain MRI (3 yrs)	Brain MRI (6 mo)	Brain MRI (18 mo)	Brain MRI (3 yrs)
Neuroimaging findings Cerebral atrophy	+	+	+	+	N/A	+	+	−	+	−	+	+
Hypomyelination	+	−	+	+	N/A	+	+	−	+	+	+	−
Corpus callosum thinning	+	+	+	+	N/A	N/A	N/A	−	+	+	+	+
Facial dysmorphy	At 13 yrs showing prominent teeth and everted lower lip.	Facial photograph of individual II-2 (Family 1) at 10 yrs shows high forehead and hypotonic face	Facial photograph of individual II-1 (Family 2) at 15 yrs shows a wide mouth	Facial photograph of individual II-2 (Family 2) at 10 yrs shows a wide mouth.	−	Facial features of individual II-6 at 6 yrs showing thick lips, crowded teeth, low frontal Hairline, remarkable nose, and bulbous nasal tip.						Frontal hump, convergent strabismus of the eye, globular eyes, incisors large and prominent, prominent upper dental arch

F, female; M, male; N/A, not available; SD, standard deviation; d, day; mo, Months; yrs, years; GTC, generalized tonic-clonic; w, weeks; DD/ID, Developmental delay/intellectual disability

In addition to this phenotypic heterogeneity associated to GRM7 mutations described in the Marafi et al. series, an allelic variability was also noticed [7]. In fact, these mutations could be located anywhere in the gene including the transmembrane domain and the ligand-binding domain [7]. In the present study, the c.1411G>A p. (Gly471Arg) variant is located in the conserved VFTD N-terminal sub-domain of the protein which plays a crucial role in glutamate binding [9, 26]. According to the bioinformatic tools, the p.Gly471Arg missense variant leads to conformational changes of the N-terminal domain and the modification of hydrogen bonds that probably disturb the mGlu7 protein folding and stability. Furthermore, docking analysis performed in the large cavity that forming the glutamate-binding site in the mutated protein showed that the p.Gly471Arg variant caused complete burial of ligand inside the binding pocket of the mGlu7 protein. Indeed, the replacement of hydrophobic (Glycine) by hydrophilic (Arginine) amino acid caused by the p.Gly471Arg variant might decrease glutamate binding [27]. Thus, based on our bioinformatic and docking analyses and according to recently reported functional studies, we suggest that the misfolded mGlu7-Gly471Arg protein might be degraded via the proteasomal and/or autophagosomal-lysosomal pathway. Indeed, functional studies were performed on mGlu7 proteins mutated in the VFTD extracellular dimerization domain [28, 29], comparable with the mutation observed in our case. These studies showed that the p.I154T mutation disrupted the mGlu7 receptor dimerization, caused a post-transcriptionally reduced expression level of mGlu7 I154T and impaired its trafficking towards the neuronal cell surface to bind to the ligand [28, 29].

The binding of glutamate to the VFTD domain is crucial to initiating conformational changes through extracellular cysteine-rich domains (N-terminal domain) and then in the transmembrane and cytoplasmic domains of the mGlu7 receptor leading to correct synaptic transmission [9, 26]. In fact, the c.1411G>A (p.Gly471Arg) mutation in the N-terminal domain of the mGlu7 could therefore disturb the signaling pathway and subsequently alter synaptic transmission. This is consistent with the reported results of Song et al. who demonstrated a marked decrease of the axon outgrowth of *GRM7* variants in the primary cultures of neurons compared to WT, consequently leading to a decrease of presynaptic terminations in mature neurons [28]. On the other hand, Fisher et al. investigated the mechanistic links between mutations located in the VFTD domain of mGlu7 and the NDD phenotypes using mGlu7-I154T knock in mice. Indeed, GRM7^I154T/I154T^ mice exhibited a significant loss in body weight, locomotor disorders, convulsive seizures, and brain weight and corpus callosum reductions [29]. Overall, these clinical and functional data demonstrate that mutations in the VFTD domain of the mGlu7 receptor should be considered as a potential cause of developmental and epileptic encephalopathy disease phenotypes.

In conclusion, we identified a novel homozygous missense mutation c.1411G>A (p.Gly471Arg) in the *GRM7* gene segregating with the disease in a large consanguineous Tunisian family comprising several cases of developmental and epileptic encephalopathy. Bioinformatic analysis supports the pathogenicity of the variant and docking analysis revealed its potential effects on mGlu7 protein binding to its ligand.

## Data Availability

Not applicable (Our manuscript does not contain any data which cannot be shared).
